# Social avoidance and social adjustment in Chinese preschool migrant children: the moderating role of teacher–child relationships

**DOI:** 10.3389/fpsyt.2023.1149319

**Published:** 2023-06-02

**Authors:** Jingjing Zhu, Xiaoqi Yin, Xiaoyun Li, Xinyi Dong, Shiyao Zou, Yan Li

**Affiliations:** Early Childhood Education College, Shanghai Normal University, Shanghai, China

**Keywords:** social avoidance, teacher–child relationships, preschool migrant children, social adjustment, China

## Abstract

**Objectives:**

This study aimed to explore the moderating role of teacher–child relationships in the relations between social avoidance and social adjustment (i.e., prosocial behavior, peer exclusion, and anxious-fearful behavior) in Chinese migrant preschoolers.

**Methods:**

Participants were 148 migrant children aged 4–6 years (82 boys, *M*_age_ = 62.32, *SD* = 6.67) attending kindergartens in Shanghai, People's Republic of China. Mothers reported children's social avoidance, and teachers rated teacher–child relationships and children's social adjustment.

**Results:**

Results indicated that social avoidance was positively related to peer exclusion and negatively related to prosocial behavior. Teacher–child relationships moderated those associations. Specifically, teacher–child closeness buffered the relationship between social avoidance and peer exclusion, whereas teacher–child conflict exacerbated the relations between social avoidance and peer exclusion and anxious-fearful behavior.

**Conclusion:**

The current finding informs us of the importance of improving teacher–child closeness and reducing teacher–child conflict to buffer the negative adjustment among socially avoidant young children who migrated from rural-to-urban China. The findings also highlight the importance of considering the meaning and implication of social avoidance for migrant preschoolers in Chinese culture.

## Introduction

Peer interactions play a crucial role in children's social status and their smooth school adjustment (1). Accordingly, socially avoidant children who frequently refuse to engage in social interactions and seek to stay alone are more likely to miss the opportunities to develop positively (2, 3) and exhibit widespread social adjustment difficulties (e.g., internalizing problems and peer problems) (4, 5). Nevertheless, some factors may exacerbate or buffer socially avoidant children from social difficulties (6). Indeed, teacher–child relationships may influence children's developmental outcomes in kindergarten settings, including peer interactions (7, 8). Furthermore, in China, the remarkable increase in economically driven rural-to-urban migration has led to a concomitant influx of rural children into the cities, forming a group of migrant children (9), who are more likely to face enormous challenges and suffer from various adjustment problems (10, 11). Moreover, teacher–child relationship status was worse for migrant children, which might be associated with more negative outcomes (12, 13). In the present study, we explored the potential moderating role of teacher–child relationships in the social adjustment of socially avoidant children in early childhood who migrated from rural-to-urban areas in China.

## Overview of social avoidance in childhood

The children who frequently tend to remove themselves from social interactions were described as socially withdrawn and more likely to miss out on opportunities to learn from the social context (3). Social withdrawal is a multi-dimensional construct that includes shyness, unsociability, and social avoidance, which reflects different underlying motivational substrates (2, 3, 14). Specifically, *shy* children want to socialize with peers but show withdrawal behavior because of fearfulness and social evaluation anxiety (2, 15). In Western culture, extensive literature proved that shyness was associated with a range of adjustment difficulties from early childhood to adolescents, such as negative peer experience (e.g., rejection and victimization) and internalizing problems (e.g., loneliness, depression, and anxiety) (5, 16). While children considered *unsociable* usually have no interest in social activities and prefer to play alone, they would not actively refuse to interact with others (2, 17). Unsociability has been viewed as relatively benign in Western culture, which encourages personal choice and autonomy (18, 19). Unlike shyness, unsociability was not associated with the indices of peer difficulties and internalizing problems (16, 20).

In the current study, we focus on *social avoidance*, characterized by actively escaping social interactions and preferring to stay alone (2, 14). It should be noted that researchers pointed out that socially avoidant children may face the greatest risk of social and emotional difficulties (2, 16). Many previous studies have confirmed this standpoint that compared to shyness and unsociability, social avoidance was associated with more adjustment difficulties at various development stages, such as peer difficulties and internalizing problems (5, 21, 22). For example, Coplan et al. (21) found that in a sample of Canadian children (aged 9–12), compared to shyness and unsociability, socially avoidant children reported the highest scores on social anxiety and depression (21). A study conducted among American school-aged children found that peer-identified avoidant children were more likely to be disliked and faced more peer exclusion and victimization (22). Furthermore, Coplan et al. found that social avoidance was a significantly greater unique predictor of peer problems than shyness, but unsociability was not a significant predictor of peer problems among preschoolers in Canada (16). Therefore, based on the existing literature, it is known that social avoidance generally has a unique predictive effect on social maladjustment in various development stages.

## Social avoidance in China

Due to culturally diverse backgrounds, social avoidance varies in its influence mechanism (23). In Western individualistic societies, withdrawing from the peer group may be seen as an expression of personal habits and autonomy (9).

Withdrawing from the peer group can be caused by many reasons in Western societies. For example, a longitudinal study highlighted the central role of negative peer relationships in the development course of social withdrawal during late childhood and early adolescence (24). Additionally, reduced family cohesion and increased parental conflict can lead to children's social withdrawal behavior (25). However, this individualistic preference to detach from the group and avoid social interaction may be considered negative in China, where social norms emphasize group harmony and cohesion, and encourage children to make friends and initiate social interactions (9, 26). A host of studies have supported this view, which consistently found that shy and unsociable children displayed internalizing problems and peer difficulties in urban China (4, 27).

Compared to shyness and unsociability, empirical studies of social avoidance were relatively limited in China, and the limited existing evidence revealed that social avoidance was also associated with more negative outcomes and maladjustment (e.g., peer exclusion, loneliness, academic outcomes, and anxious-fearful behavior) in various development stages (4, 6, 27), which may be the greatest risk subtype of social withdrawal (2, 16). For example, Sang et al. reported that, compared to shyness and unsociability, social avoidance had unique relationships with internalizing and peer problems among Chinese young adolescents (28). In addition, Ding et al. found that, compared to the hypothetical shy and unsociable peers, Chinese children in kindergarten and grade 1 anticipated that the hypothetical avoidant peers might have the most negative outcomes (26).

With the implementation of a full-scale market economy reform, China's society has undergone dramatic changes over the past years. Due to the changes in external factors such as living environment and various factors at the individual level, migrant children are often faced with huge challenges (11, 29–31). Studies have shown that compared with urban non-migrant children, migrant children have poorer academic performance and are more likely to suffer from various psychological problems such as inferiority complex, depression, and loneliness (11, 28, 32). Additionally, due to unfamiliar surroundings, migrant children are more likely to suffer from social anxiety in peer interactions (33). Therefore, Chinese migrant children who avoid social interaction may face the risk of being rejected and hated by their peers, which in turn makes it difficult for them to establish close peer relationships (16, 34).

On this background, it may be argued that adjustment difficulties continue to be elevated among migrant children, compared with non-migrant children, which may be reflected in the experiences of socially avoidant children. However, most studies on social avoidance and its adjustment were conducted on non-migrant children in Chinese cities. To the best of our knowledge, only minimal research has been carried out on social withdrawal and social adjustment in the particular group of migrant children living in urban areas in China, and mainly focused on the subtype of shyness and unsociability (9, 35). Indeed, migrant children were more likely to maintain the traditional social behaviors valued in past China and children who were behaviorally inhibited and self-restrained were encouraged (19). Nevertheless, new behavioral characteristics, including cooperation and self-expression were increasingly encouraged in the competitive urban environment (36). Thus, the adjustment pressure of socially avoiding migrant children may be stronger in the new social requirements in urban China. Thus, exploring the social adjustment of socially avoidant migrant children in China is necessary, which can expand the research on social avoidance in Chinese migrant children. Furthermore, compared to school-age migrant children, preschool migrant children (aged 0–5) accounted for almost one-third of the total migrant children in China (37) and have been persistently neglected in previous literature (38). However, preschool migrant children are at a critical stage of individual development, when they might be more sensitive to stressful environments.

Given the lack of existing empirical evidence on social avoidance and adjustment in early childhood among Chinese migrant children, one goal of the present study was to explore the implications of social avoidance in a sample of Chinese migrant preschoolers.

## The moderating role of teacher–child relationships

Given that avoidant migrant preschoolers in China may experience extensive social maladjustment, it is essential to identify the underlying moderating factors that may exacerbate or buffer the adjustment outcomes. It may further help the design of prevention and intervention programs for avoidant migrant preschoolers in China. The present study examined teacher–child relationships as the potential moderating factor between social avoidance and adjustment.

Children in their early years usually face the transition from family to kindergarten (8). According to attachment theory, preschool teachers serve as temporary attachment objects; in addition to playing the role of educator, they also play the role of caregiver in interactions with young children, and the nature of interaction directly affects the establishment of a safe emotional connection between preschool teachers and young children, which was similar to the parent–child relationship (8, 39). Thus, harmonious teacher–child relationships may have positive effects on children's development.

Researchers often measure the levels of closeness and conflict in teacher–child relationships (40–42). Specifically, *teacher–child closeness* referred to warm and open communication between teachers and their children and was associated with more positive developmental outcomes for children, such as more effective social-emotional skills and better academic skills (8, 43, 44). For example, Hartz et al. (45) revealed that positive and close relationships between teachers and preschoolers were associated with peer interactions. On the contrary, *teacher–child conflict* was manifested in the negative and highly tense relationships between teachers and their children and was associated with more negative developmental outcomes, such as lower academic achievement, more externalizing behaviors, and loneliness (46–48). For example, Li et al. found that teacher–child conflict in kindergarten related to future academic skills in primary school (40). In addition, Saral and Acar found that teacher–child closeness was positive and teacher–child conflict was negatively associated with preschool children's social competence (42). Higher levels of teacher–child closeness would mitigate the negative effect of parent–parent conflict on children's social competence (42). Thus, it seems reasonable to consider the role of teacher–child relationships, in the link between social avoidance and social adjustment in migrant preschoolers.

According to the *Diathesis Stress Model*, children with “risk” diathesis, such as children with social avoidance, are more likely to have adjustment difficulties or psychological disorders when encountering unfavorable external environments (49).

Socially avoidant children tend to be more sensitive and reactive to social stimuli, and they may have difficulty making friends or participating in group activities (50). This heightened sensitivity to social situations may make socially avoidant children more susceptible to negative experiences. Research has found that socially avoidant children experienced more negative outcomes, such as increased anxiety and peer exclusion when exposed to more maternal psychological control (51). Moreover, studies have indicated teacher–child conflict's risk role in exacerbating social adjustment difficulties (41, 42). Indeed, a previous study has shown that when children are exposed to more teacher–child conflict, they exhibit elevated internalizing and externalizing problems (52). Similarly, Zhu et al. (53) suggested that children with high teacher–child conflict had higher levels of behavior problems relative to other children. Therefore, when socially avoidant children experience a high level of teacher–child conflict, it increases the risk of children's social maladjustment (53).

However, socially avoidant children may also benefit more from a positive childcare environment, such as low levels of household chaos having a buffering influence on socially avoidant children's risk for interpersonal skills (6). In fact, teacher–child closeness has been demonstrated as a protective factor for children's social maladjustment (46, 54, 55). For example, Coplan et al. reported that at higher levels of teacher–child closeness, the relation between shyness and peer preference was attenuated in a sample of young Chinese children (46). In addition, the findings of a prospective cohort study with 7,343 preschool children suggested that shy children's risk for social difficulties can be mitigated by early teacher–child closeness (54). Furthermore, the *Attachment Theory* points out that teachers are important attachment objects and the safe harbor of children (56, 57). In this sense, when socially avoidant migrant preschoolers had positive relationships with their teachers, they would benefit from the supportive environment provided by teachers, which in turn leads to better social adjustment (54, 58).

To summarize, the association between social avoidance and social adjustment may be moderated by teacher–child relationships. Most existing evidence showed that children's social adjustment varies with teacher–child relationships. However, few studies has examined the moderating role of teacher–child relationships in the relationship between social avoidance and social adjustment in Chinese culture, not to mention among Chinese migrant children. Thus, in the present study, we examined the moderating role of teacher–child relationships between social avoidance and social adjustment in a sample of Chinese migrant preschoolers.

## The present study

As mentioned above, social avoidance and teacher–child relationships were associated with social difficulties among Chinese young children, such as peer difficulties, internalizing and externalizing problems, and social anxiety (6, 8). However, to date, nearly no study has explored the underlying mechanism of teacher–child relationships in the relationships between social avoidance and social adjustment in Chinese preschoolers, not to mention among the migrant preschoolers who are likely to experience worsened adjustment difficulties (10–12). Therefore, drawing upon the extant literature, we focused on three main aspects of social adjustment: prosocial behavior, peer exclusion, and anxious-fearful behavior.

**Figure 1:**
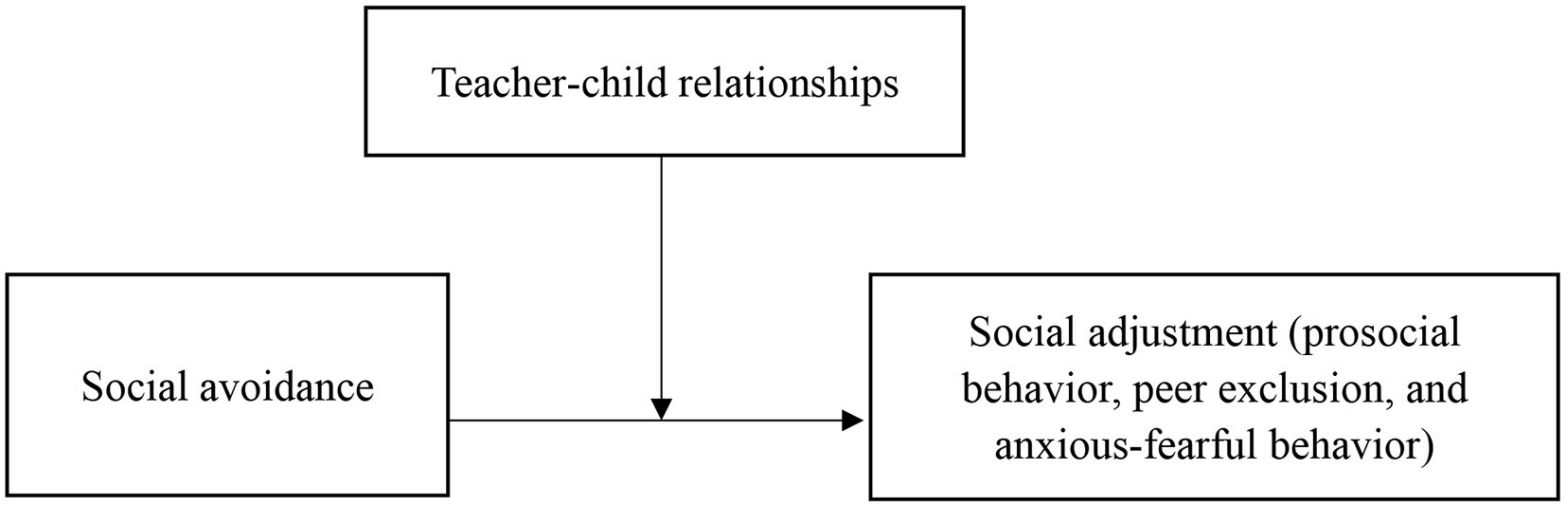
Hypothesis model.

In summary, the principal purpose of this study was to extend previous research by investigating the moderating role of teacher–child relationships (i.e., teacher–child closeness and teacher–child conflict) in the relationship between social avoidance and social adjustment (i.e., prosocial behavior, peer exclusion, and anxious-fearful behavior) among Chinese preschool migrant children. We hypothesized that social avoidance would be positively associated with peer exclusion and anxious-fearful behavior, while social avoidance was negatively associated with prosocial behavior. Moreover, in terms of the moderating effect of teacher–child relationship, we assumed that negative associations between social avoidance and social adjustment would be weaker among migrant children with higher levels of teacher–child closeness and stronger among those with higher levels of teacher–child conflict (see Figure 1).

## Method

### Participants

Participants consisted of 148 migrant children (82 boys, 66 girls, *M*_age_ = 62.32 months, *SD* = 6.76) recruited from two public kindergartens in Shanghai, People's Republic of China. The two kindergartens are “mixed kindergartens,” where the migrant preschoolers attend kindergartens together with their non-migrant urban peers. In China, children attend kindergarten for 3 years and are grouped by age (e.g., juniors are 3–4 years old, middles are 4–5 years old, and seniors are 5–6 years old). All children were of Han ethnicity in this study, which makes up over 97% of China's population.

Nearly 22% of the mothers and 24% of the fathers had completed high school; 40% of the mothers and 27% of the fathers had completed junior college; 35% of the mothers and 41% of the fathers had earned a bachelor's degree; and 3% of the mothers and 7% of the fathers had earned a postgraduate degree. Maternal and paternal scores were averaged to create a broader measure of parental education (with higher scores representing higher education).

### Procedure

The present study was reviewed and approved by the ethics review board of BLIND FOR PEER REVIEW. Every child in each of the participating classes was invited to take part in the study. The school obtained written permission from the parents of all children. Our study had 98% written consent. Mothers rated their children's social avoidance and teachers completed measures of children's social adjustment and teacher–child relationships.

### Measures

#### Maternal ratings

Mothers completed the Chinese version of the Child Social Preference Scale (CSPS) (16). One of the most interesting subscales was the one assessing social avoidance, which consists of four items (e.g., “If giving a choice, my child prefers to play alone than with other kids”; α = 0.76). In exploring the implications of social avoidance among Chinese migrant children, it is imperative to control for any associated variance with shyness and unsociability due to the conceptual overlaps and similar patterns of adjustment (28). As such, mothers also completed the *shyness* subscale, which comprises seven items (e.g., “Although he/she appears to desire to play with others, my child is sometimes anxious about interacting with other children”; α = 0.89), and *unsociability* subscale, which comprises four items (e.g., “My child is just as happy to play quietly by his/herself than to play with a group of children”; α = 0.67) rated on a 5-point Likert-type scale (from 1 = “not at all” to 5 = “a lot”). Higher scores for CSPS-3 subscales indicated higher levels of social avoidance, shyness, and unsociability, respectively. The CSPS has demonstrated good reliability and validity in Chinese children (59, 60).

#### Teacher ratings

Teachers completed the Chinese version of the Student–Teacher Relationship Scale (STRS) (61, 62). In this study, we focused on conflict (12 items, e.g., “This child and I always seem to be struggling with each other”; α = 0.84) and closeness (11 items, e.g., “I share an affectionate, warm relationships with this child”; α = 0.85) scales of the STRS to measure teacher–child relationships. Items were rated on a 5-point Likert-type scale (from 1 = “definitely does not apply” to 5 = “definitely applies”). The STRS showed reliability and validity in young Chinese children (62).

Teachers also completed the Chinese version of the Child Behavior Scale (CBS) (63, 64). One of the most interesting subscales was the one assessing prosocial behavior (seven items, e.g., “often help”; α =0.90), peer exclusion (seven items, e.g., “not welcomed by other children”; α = 0.86), and anxious-fearful behavior (four items, e.g., “Poor concentration, attention span”; α =*0*.73). Items were rated on a 3-point Likert-type scale (from 1 = “doesn't apply” to 3 = “certainly applies”). The CBS has demonstrated good reliability and validity in Chinese children (64).

### Analytical strategy

We used SPSS 24.0 software for data analysis. Aiming to explore gender differences and correlations among study variables, we used a series of *t*-tests in preliminary analyses. Then we used the PROCESS macro (65) (Model 1) with non-parametric bootstrapping with 5,000 resamples to explore the moderating effect of teacher–child relationships between social avoidance and indices of social adjustment. For the test of significant interactions, we used a 95% bias-corrected confidence interval (CI) (66). The moderating effect was thought to be significant when the zero was not included in the 95% bias-corrected confidence interval (CI) of an interaction term (social avoidance × teacher–child relationships) (66). For the significant two-way interactions, we further conducted simple slope analyses and plotted the relationships between social avoidance and social adjustment variables in a high value (+1 SD above the mean) and a low value (-1 SD below the mean) of teacher–child relationships (67). In addition, in order to probe for significant regions of social avoidance on adjustment variables at different values of teacher–child relationships, each mother's ratings and teacher's ratings of each scale were transferred to z-scores, and then the Johnson–Neyman (J–N) technique (68) was conducted.

## Results

### Preliminary analyses

Results from *t*-tests indicated that there were significant gender differences in teacher–child conflict (*M*_boy_ = 1.56, *SD* = 0.65; *M*_girl_ = 1.36, *SD* = 0.42, *t* = 2.23, *p* = 0.03), teacher–child closeness (*M*_boy_ = 3.65, *SD* = 0.75; *M*_girl_ = 3.92, *SD* = 0.68, *t* = −2.31, *p* = 0.02), prosocial behavior (*M*_boy_ = 2.25, *SD* = 0.57; *M*_girl_ = 2.45, *SD* = 0.52, *t* = −2.30, *p* = 0.02), and peer exclusion (*M*_boy_ = 1.23, *SD* = 0.43; *M*_girl_ = 1.07, *SD* = 0.20, *t* = 2.72, *p* = 0.01). Gender differences had no effect on other variables. Accordingly, we controlled for child gender in subsequent analyses.

As shown in Table 1, child age was positively correlated with teacher–child closeness. Social avoidance was significantly and positively associated with peer exclusion, and negatively associated with prosocial behavior. Teacher–child conflict was also significantly and positively associated with peer exclusion and anxious-fearful behavior and negatively associated with prosocial behavior. Teacher–child closeness was significantly and negatively associated with peer exclusion and anxious-fearful behavior, and positively associated with prosocial behavior.

**Table 1:** Descriptive statistics and Spearman correlations among all study variables (*N* = 148).

**Variables**	**1**	**2**	**3**	**4**	**5**	**6**	**7**	**8**	**9**	**10**	**11**
1. Gender	-										
2. Age (month)	−0.06	-									
3. Parental education	0.02	0.04	-								
4. Shyness	0.01	−0.07	0.02	-							
5. Unsociability	−0.08	−0.10	0.07	0.63^***^	-						
6. Social avoidance	0.06	−0.10	0.07	0.57^***^	0.62^***^	-					
7. Teacher**–**child conflict	−0.18^*^	0.06	−0.03	0.04	0.14	0.05	-				
8. Teacher**–**child closeness	0.19^*^	0.36^***^	0.04	−0.31^***^	−0.33^***^	−0.21^*^	−0.38^***^	-			
9. Prosocial behavior	0.19^*^	0.46^***^	0.10	−0.27^***^	−0.29^***^	−0.20^*^	−0.42^***^	0.72^***^	-		
10. Peer exclusion	−0.22^**^	0.01	0.04	0.20^*^	0.25^**^	0.23^**^	0.062^***^	−0.49^***^	−0.51^***^	-	
11. Anxious-fearful behavior	−0.05	−0.02	0.11	0.08	0.07	0.11	0.23^**^	−0.20^*^	−0.27^***^	0.41^***^	-
*M*	-	62.32	-	1.84	1.72	1.33	1.47	3.77	2.34	1.16	1.23
*SD*	-	6.76	-	0.68	0.57	0.48	0.56	0.73	0.56	0.36	0.36

* *p* < 0.05.

** *p* < 0.01.

*** *p* < 0.001.

### Social avoidance, teacher–child relationships, and social adjustment

The goal of the following analyses was to examine the moderating role of teacher–child relationships (i.e., teacher–child closeness and teacher–child conflict) in the relations between social avoidance and social adjustment (i.e., prosocial behavior, peer exclusion, and anxious-fearful behavior) while controlling for gender, age (only when the moderating variable was teacher–child closeness), shyness, and unsociability. Classroom intraclass correlations (ICCs) for all variables were <0.04, indicating no cluster effects in the classroom. The results of the regressions are shown in Table 2.

**Table 2:** Effects of social avoidance, teacher–child relationships in relation to indices of social adjustment.

**Predictor**	***B***	***SE***	***t*-value**	**95% CI**
**Social adjustment variables**				
**Peer exclusion**				
Social avoidance	0.15^+^	0.08	1.78	[−0.02, 0.31]
Teacher**–**child conflict	0.57	0.06	8.86^***^	[0.44, 0.70]
Avoidance × teacher**–**child conflict	0.20	0.06	3.18^**^	[0.07, 0.32]
**Prosocial behavior**				
Social avoidance	−0.02	0.10	−0.18	[−0.21, 0.18]
Teacher**–**child conflict	−0.38	0.08	−4.92^***^	[−0.53, −0.23]
Avoidance × teacher**–**child conflict	−0.09	0.07	−1.18	[−0.23, 0.06]
**Anxious-fearful behavior**				
Social avoidance	0.11	0.11	0.98	[−0.11, 0.32]
Teacher**–**child conflict	0.21	0.08	2.53^*^	[0.04, 0.38]
Avoidance × teacher**–**child conflict	0.20	0.08	2.49^*^	[0.04, 0.36]
**Peer exclusion**				
Social avoidance	0.16	0.10	1.64	[−0.03, 0.35]
Teacher**–**child closeness	−0.47	0.09	−5.35^***^	[−0.64, −0.29]
Avoidance × teacher**–**child closeness	−0.09	0.05	−1.76^+^	[−0.19, 0.01]
**Prosocial behavior**				
Social avoidance	−0.03	0.08	−0.34	[−0.18, 0.13]
Teacher**–**child closeness	0.59	0.07	8.42^***^	[0.45, 0.72]
Avoidance × teacher**–**child-closeness	0.00	0.04	0.04	[−0.08, 0.08]
**Anxious-fearful behavior**				
Social avoidance	0.12	0.11	1.08	[−0.10, 0.35]
Teacher**–**child closeness	−0.21	0.10	−2.08^*^	[−0.42, −0.01]
Avoidance × teacher**–**child closeness	−0.02	0.06	−0.32	[−0.14, 0.10]

Gender, age (only when the moderating variable was teacher–child closeness), shyness, and unsociability were controlled in the analysis.

* *p* < 0.05.

** *p* < 0.01.

*** *p* < 0.001.

+ p < 0.01.

Results indicated that there were significant interaction effects between social avoidance and teacher–child conflict in relation to peer exclusion and anxious-fearful behavior. Furthermore, interaction effects between social avoidance and teacher–child closeness in relation to peer exclusion were significant (marginal significance). However, there were no significant interaction effects between social avoidance and teacher–child relationships (both teacher–child closeness and teacher–child conflict) found in relation to prosocial behavior.

**Figure 2:**
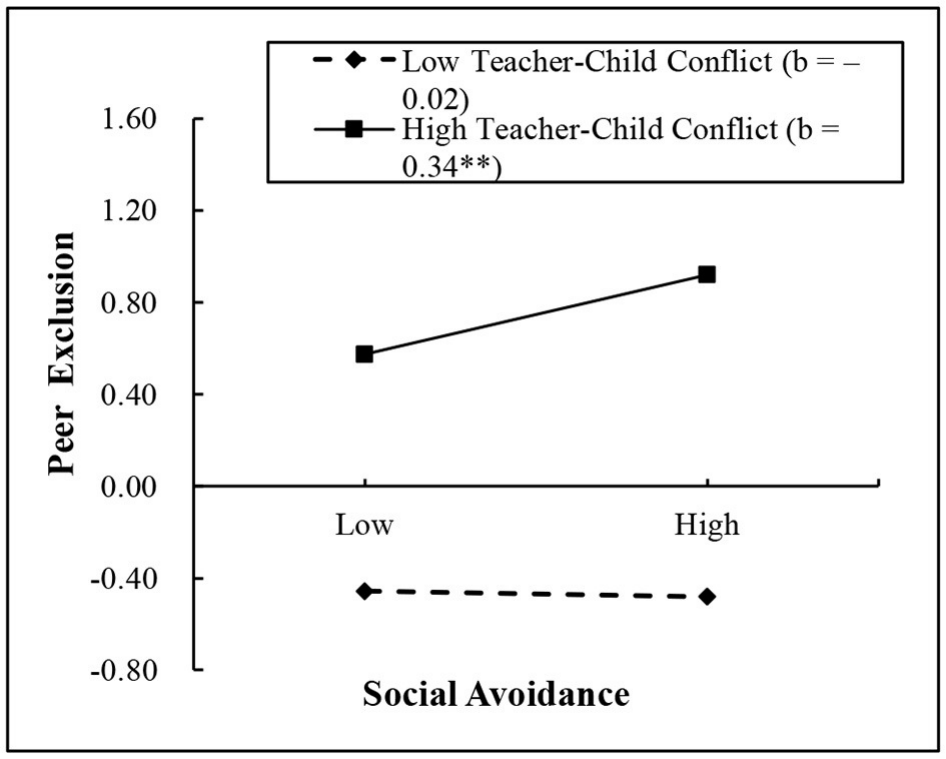
Interaction between teacher–child conflict and social avoidance on peer exclusion. ^**^p < 0.01.

**Figure 3:**
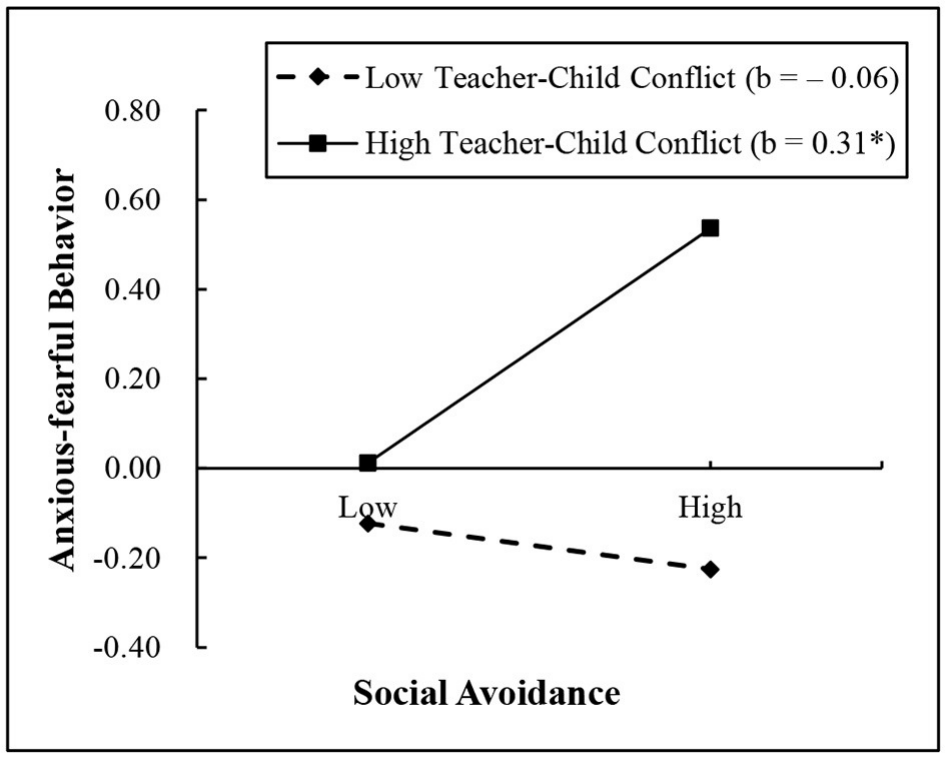
Interaction between teacher–child conflict and social avoidance on anxious-fearful behavior. ^*^p < 0.05.

Furthermore, in order to explain these significant two-way interactions, the simple slope effects were conducted on social avoidance at high and low values (1 SD above and 1 SD below the mean) of teacher–child conflict and closeness (67). The results of simple slopes are shown visually in Figures 2, 3. As shown in Figure 2, social avoidance had a positive association with peer exclusion for children with high teacher–child conflict (*b* = 0.34, *SE* = 0.10, *t* = 3.40, *p* < 0.01); on the contrary, this association was not significant for children with low teacher–child conflict (*b* = −0.02, *SE* = 0.10, *t* = −0.15, *p* > 0.05). As shown in Figure 3, social avoidance had a positive association with anxious-fearful behavior for children with high teacher–child conflict (*b* = 0.31, *SE* = 0.13, *t* = 2.32, *p* < 0.05); on the contrary, this association was not significant for children with low teacher–child conflict (*b* = −0.06, *SE* = 0.13, *t* = −0.47, *p* > 0.05). However, for the significant (marginal significance) interaction effects between social avoidance and teacher–child closeness in relation to peer exclusion, no results were found in the simple slope analysis.

Johnson–Neyman (J–N) technique was also conducted to probe the significant regions for significant interactions, as suggested by Hayes and Matthes (69).

**Figure 4:**
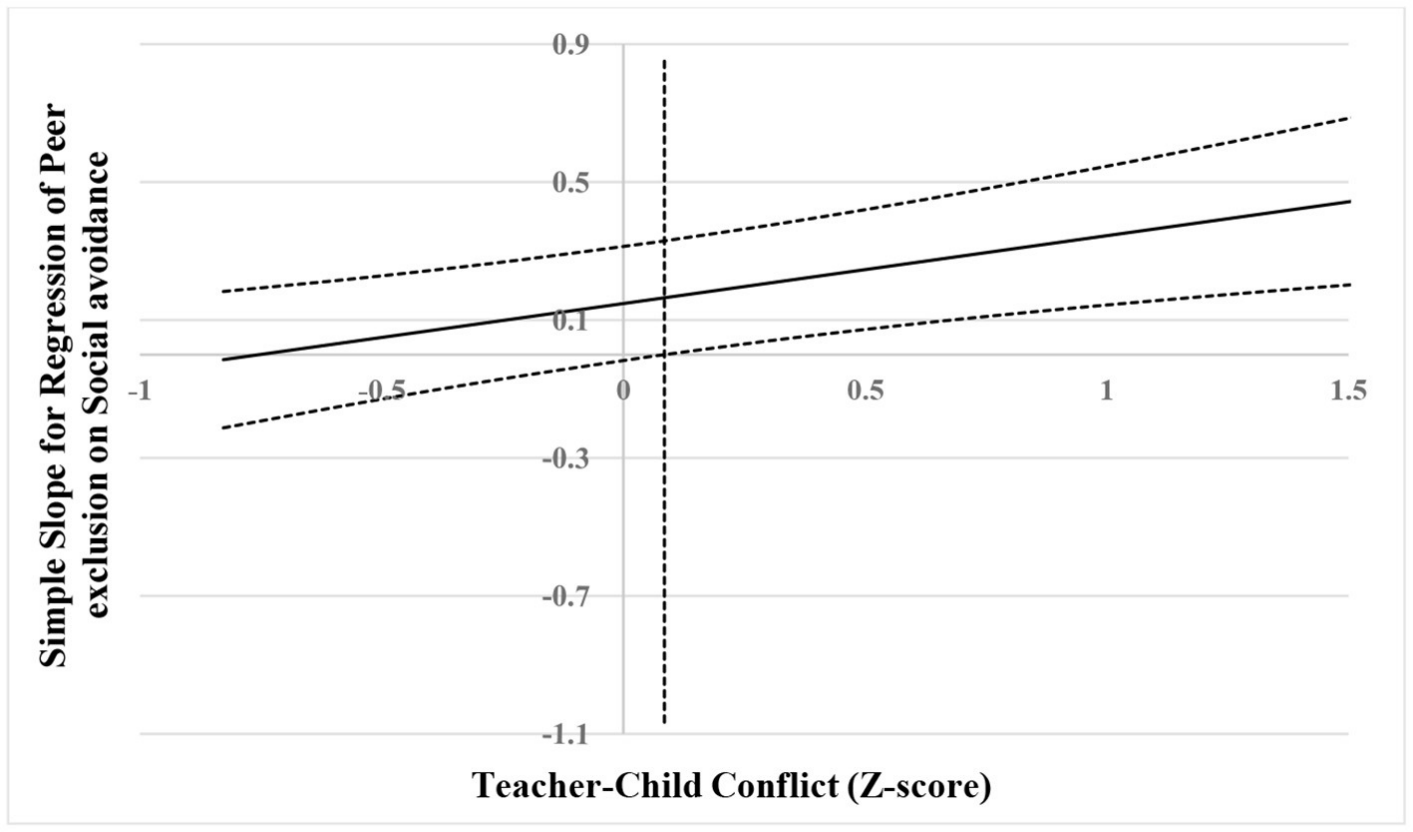
Johnson–Neyman regions of significance and confidence bands for mother-rated social avoidance along teacher-rated teacher–child conflict in relation to peer exclusion. Solid diagonal line represents the regression coefficient for social avoidance along teacher–child conflict. Dashed diagonal blue lines are confidence bands—upper and lower bounds of 95% confidence interval for social avoidance regression coefficient along teacher–child conflict. The vertical red dashed line indicates the point along teacher–child conflict at which the social avoidance regression coefficient transitions from non-significance (left of vertical dashed line) to statistical significance (right of vertical dashed line). The value of the vertical red dashed line is 0.08.

**Figure 5:**
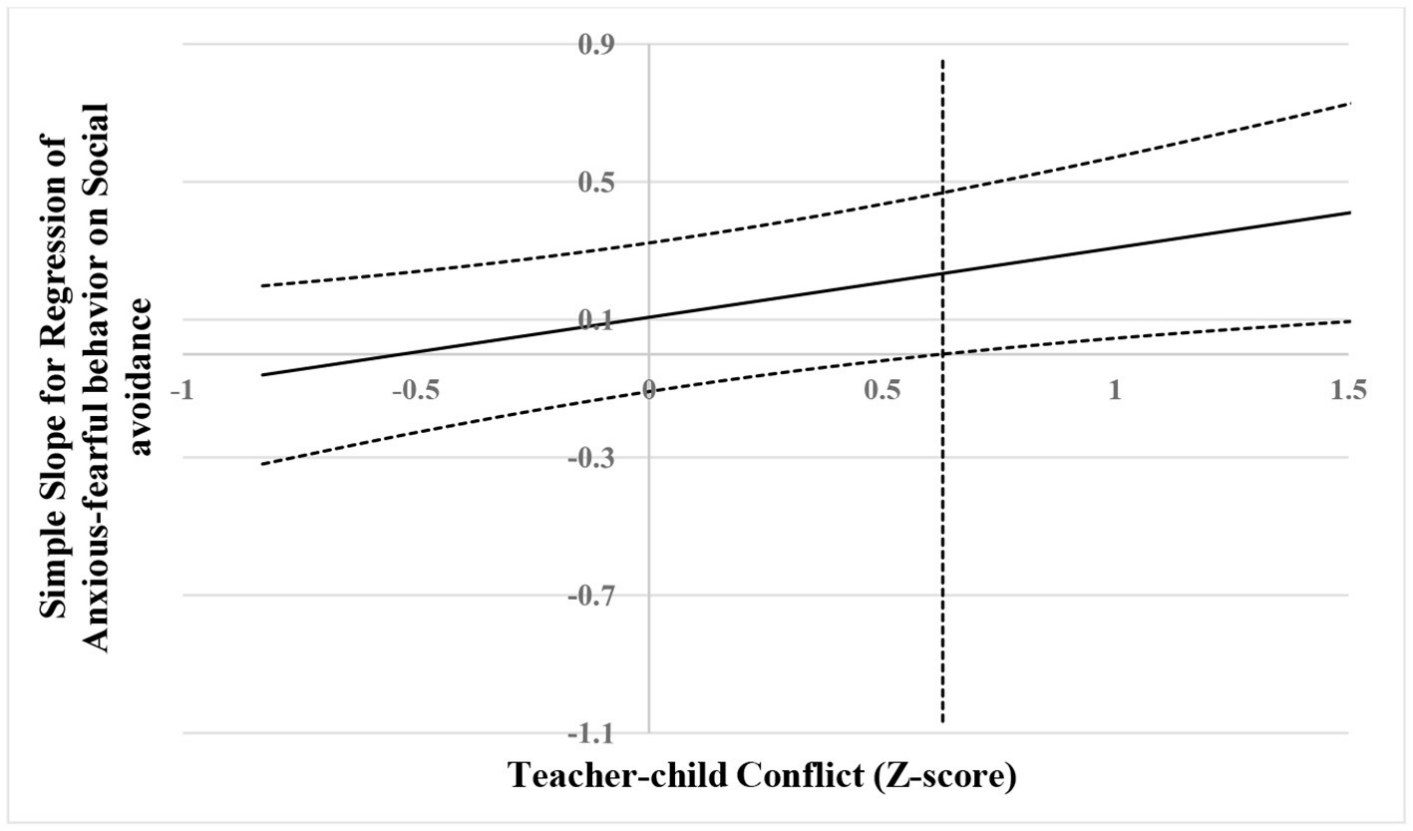
Johnson–Neyman regions of significance and confidence bands for mother-rated social avoidance along teacher-rated teacher–child conflict in relation to anxious-fearful behavior. The solid diagonal line represents the regression coefficient for social avoidance along teacher–child conflict. Dashed diagonal blue lines are confidence bands—upper and lower bounds of 95% confidence interval for social avoidance regression coefficient along teacher–child conflict. The vertical red dashed line indicates the point along teacher–child conflict at which the social avoidance regression coefficient transitions from non-significance (left of vertical dashed line) to statistical significance (right of vertical dashed line). The value of the vertical red dashed line is 0.63.

**Figure 6:**
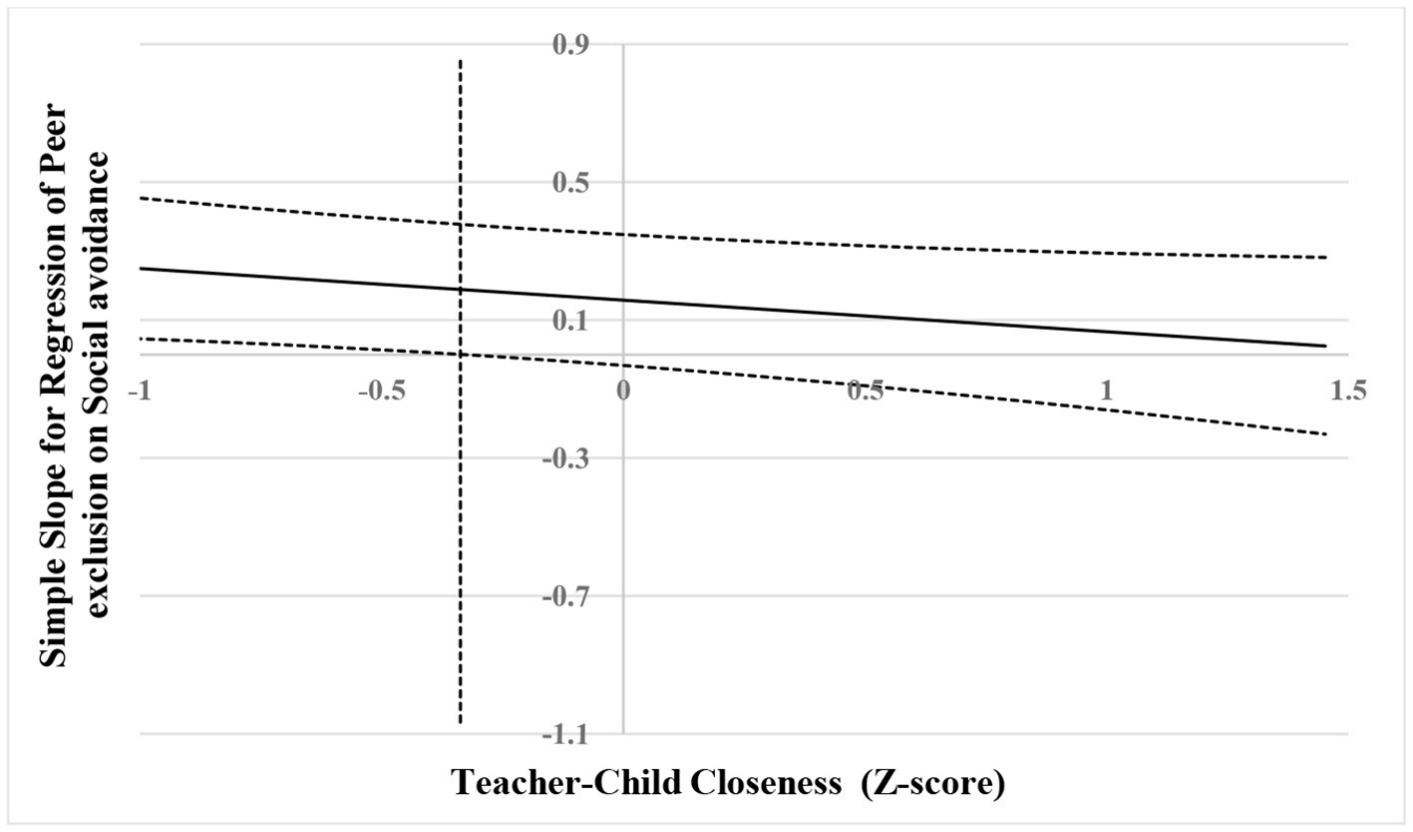
Johnson–Neyman regions of significance and confidence bands for mother-rated social avoidance along teacher-rated teacher–child closeness in relation to peer exclusion. The solid diagonal line represents the regression coefficient for social avoidance along teacher–child closeness. Dashed diagonal blue lines are confidence bands—upper and lower bounds of 95% confidence interval for social avoidance regression coefficient along teacher–child closeness. The vertical red dashed line indicates the point along teacher–child closeness at which the social avoidance regression coefficient transitions from statistical significance (left of vertical dashed line) to non-significance (right of vertical dashed line). The value of the vertical red dashed line is −0.34.

The results are shown visually in Figures 4–6. As shown in Figure 4, when the score of teacher–child conflict was higher than 0.08 SD, social avoidance was significantly and positively associated with peer exclusion. However, when the score of teacher–child conflict was lower than 0.08 SD, social avoidance was no longer associated with peer exclusion. As shown in Figure 5, when the score of teacher–child conflict was higher than 0.63 SD, social avoidance was significantly and positively associated with anxious-fearful behavior. However, when the score of teacher–child conflict was lower than 0.63 SD, social avoidance was no longer associated with anxious-fearful behavior. As shown in Figure 6, when the score of teacher–child closeness was lower than −0.34 SD, social avoidance was significantly and positively associated with peer exclusion (marginal significance). However, when the score of teacher–child closeness was higher than 0.34 SD, social avoidance was no longer associated with peer exclusion.

## Discussion

The present study expanded on previous research by examining the relations between social avoidance and social adjustment (prosocial behavior, peer exclusion, and anxious-fearful behavior) and the moderating role of teacher–child relationships in Chinese migrant preschoolers. The findings showed that social avoidance was significantly associated with prosocial behavior and peer exclusion. Moreover, teacher–child conflict exacerbated the relationship between social avoidance and migrant preschoolers' adjustment problem, whereas teacher–child closeness buffers the social adjustment difficulties of socially avoidant migrant preschoolers in China. These aspects of the results are discussed in detail below.

### Association between social avoidance and social adjustment

As expected, results suggested that social avoidance was significantly and negatively associated with prosocial behaviors and significantly and positively associated with peer exclusion. These results were consistent with previous studies that social avoidance was associated with social maladjustment in Chinese migrant preschoolers (6). However, Zhu et al. found that social avoidance was not significantly associated with peer exclusion among non-migrant preschoolers living in urban areas in China (60). Thus, as compared to socially avoidant non-migrant preschoolers, socially avoidant migrant preschoolers might have poorer social skills and face more peer difficulties.

Based on *Embedding and Disembodying Theory* (70), migrant children were at risk of being under-embedded in the urban environment. They had to constantly face and cope with new and unfamiliar territory (71). Thus, socially avoidant migrant preschoolers were more likely to feel anxious or insecure about their interpersonal relationships (30, 31), which increased their avoidance of peer interaction. In addition, according to *Developmental Contextualism* (72), children prefer to interact with peers with similar behavior and attitude characteristics (73). However, as migrant families tend to maintain traditional socialized behavior, the attitudes and behaviors of migrant children are more like those of peers in the countryside (9, 74). Thus, it is relatively more challenging for migrant socially avoidant preschool children to receive peer acceptance.

Then, we also found that social avoidance was significantly and negatively correlated with prosocial behavior. It could be that socially avoidant migrant preschoolers tend to remain isolated from peers, and naturally, it is difficult for them to initiate prosocial interactions with others (i.e., helping and comforting).

### Moderating role of teacher–child relationships

Consistent with our hypotheses, teacher–child relationships moderated the association between social avoidance and the indexes of social adjustment.

Specifically, after controlling for shyness and unsociability, teacher–child conflict aggravated socially avoidant migrant children's peer exclusion and anxious-fearful behavior, and teacher–child closeness buffers the relationships between social avoidance and peer exclusion in Chinese migrant preschoolers.

Regarding the moderating role of teacher–child conflict, the result revealed that social avoidance was positively associated with peer exclusion and anxious-fearful behavior among migrant preschoolers with high teacher–child conflict in China, but not for the children with low teacher–child conflict. These findings align with previous findings, suggesting that higher levels of teacher–child conflict may play a dangerous role in peer interactions among socially avoidant migrant children (75). Teacher–child conflict may prevent socially avoidant migrant children from forming secure attachments with teachers. Based on the *Emotional Security Theory* (76), insecure attachment keeps children from feeling safe in kindergarten and further prompts social anxiety and fear of interpersonal communication. Moreover, socially avoidant migrant preschoolers whom teachers do not support are less likely to pay attention to teachers' behaviors, which leads to fewer opportunities for them to learn social skills (77), thus showing peer problems. The findings supported the Diathesis Stress Model, in that the sensitivity of socially avoidant migrant preschoolers to negative environments would be intensified, and when they have experienced a high level of teacher–child conflict, they might feel more stressed and exhibit peer exclusion and anxious-fearful behavior.

We believe that the results of the present study need to be understood in the context of migrant children in China. Most migrant children often follow their parents from rural areas to urban areas. Their previous social experience is guided mainly by traditional values in rural areas but by a more modern value system in cities (78). Finding a balance between urban culture and rural culture is crucial for the social and school adjustment of migrant children (79, 80). For migrant children with social avoidance, they are more likely to suffer from social maladjustment after experiencing mixed social and cultural standards and belief systems. In addition, migrant preschoolers may adopt the adult-oriented approach to socializing in the family and school, and teachers' guidance and support role in school may further affect the functional significance of social avoidance in their adjustment to a certain extent. In contrast, teacher–child conflict aggravated the socially avoidant migrant preschoolers' maladjustment.

Regarding the moderating role of teacher–child closeness, no results were found in the further simple slopes analyses. However, the results of the regions for significant interactions found in the Johnson–Neyman technique further revealed that social avoidance was positively associated with peer exclusion when migrant children reported lower teacher–child closeness. However, social avoidance had a non-significant predictive effect on peer exclusion when migrant children reported higher teacher–child closeness. These findings were consistent with previous research identifying the protective role of positive teacher–child relationships in social-emotional function and academic performance from risk factors for the children (27, 55, 75). The challenges with migration (e.g., cultural differences, adjustment to a new living environment, and insecurity) can result in more profound feelings of loneliness, social anxiety, alienation, and worthlessness (11, 29, 33), and make it difficult for migrant children who already have difficulty in interacting socially to receive support in new relationships. In fact, social support is essential for children to cope with stress and adjustment difficulties (1). Therefore, social support from teachers is an important factor in helping socially avoidant children to cope with migrantions' stress and difficulties. Moreover, establishing close relationships with teachers will increase socially avoidant migrant preschoolers' sense of security at school (81) and embolden them to explore peer relationships boldly. Furthermore, *Social Learning Theory* pointed out that teachers can shape children's social interaction behaviors (54). The migrant preschoolers who always avoid interacting with peers may learn better social skills from close interactions with teachers. It is also possible that in a classroom environment with a high level of teacher–child closeness, teachers are more likely to provide more emotional care and encouragement to socially avoidant migrant preschoolers and help them develop effective strategies to interact with peers more positively (82). Additionally, children who are close to their teachers also seem to be more receptive to and actively participate in social activities arranged by their teachers, which in turn reduces their avoidance behavior, and the relationship between peers will be further improved, and peer exclusion will be reduced in the daily interaction with peers. In brief, the current study is the first to provide preliminary evidence to suggest that although social avoidance may have negative implications for Chinese migrant preschoolers, teacher–child closeness may serve as an important protective factor.

Contrary to expectations, teacher–child relationships (both teacher–child closeness and teacher–child conflict) not significantly moderated the effects of social avoidance on prosocial behavior. Previous studies revealed that preschoolers tend to be self-centered, and they may not pay much attention to peers, solitary behavior is quite common (17). In addition, the behavior of socially avoidant preschoolers is characterized by a preference for solitude and avoidance of peer interaction, which teachers and parents may perceive as a manifestation of poor prosocial behavior. Moreover, migrant preschoolers in China generally have to face unfamiliar territory and challenging settings, and there may be many other factors related to prosocial behavior. For example, previous studies found that migrant families generally living at a lower level of socio-economic status, which led to more life stresses and conflict among family members (83), and further led to negative parent-child relationships which in turn affected migrant children's adjustment problems (83). Thus, the unique relationship between social avoidance and internalizing problems may not be exhibited in migrant preschoolers. Although these findings were unexpected, future studies exploring mechanisms underlying social avoidance are still needed to further our understanding of how (and under which circumstances) socially avoidant children may be at risk for subsequent social adjustment difficulties.

### Limitations and future direction

Several limitations to this study need to be considered. First, the study was cross-sectional, not revealing causality between variables. For example, in terms of our interpretation, it is also possible that migrant preschoolers who have poor peer relationships, in turn, increase their social avoidance. Future studies should use a longitudinal design to explore the interaction between variables better.

Second, because the preschoolers were too young to complete the written questionnaires, all data collection is based on mother and teacher reports, which may be biased. For example, researchers found an association between teachers' negative feelings about relationships with children they perceive as having negative behavior (84). Future studies should conduct multiple sources (e.g., peers and parental reports) for each variable.

Third, the sample of the present study was selected from Shanghai, which could limit generalizing the results to other areas. Future studies need to expand the sample population to better (e.g., different cultural backgrounds and greater socioeconomic diversity). Additionally, a comparison group of non-migrant children who moved into an urban area would also help future studies gain a more comprehensive perspective on the migrant acculturation experience and enhance future studies.

Fourth, we focused on socially avoidant migrant preschoolers, likely affected by contextual factors (85). Other aspects of migrant variables, such as the length of residence of migrant preschoolers in cities and the acculturation of migrant children (i.e., different cultural standards and belief systems), may serve different functions in the adjustment of children with different backgrounds (80). Further research is needed to account for the involvement of context in migrant preschoolers' social adjustment.

Despite these limitations, this research explored the relations between social avoidance and social adjustment in Chinese migrant preschoolers for the first time and found the protective role of teacher–child closeness and the risky role of teacher–child conflict in those relationships. Such findings have practical implications for early intervention programs for socially avoidant migrant preschoolers. Teachers should know that their relationships with children play an important role in migrant preschoolers' early social development. Therefore, practitioners and educators should pay attention to children who avoid social interaction, especially migrant children, give them warmth and support, and help children engage in social relationships.

## Data availability statement

The raw data supporting the conclusions of this article will be made available by the authors, without undue reservation.

## Ethics statement

The studies involving human participants were reviewed and approved by Shanghai Normal University. The patients/participants provided their written informed consent to participate in this study.

## Author contributions

JZ, XY, and XL managed the literature search and analyses. XD and SZ participated in data collection. YL designed the study. All authors contributed to the article and approved the submitted version.
